# The Regulatory Code for Transcriptional Response Diversity and Its Relation to Genome Structural Properties in A. thaliana

**DOI:** 10.1371/journal.pgen.0030011

**Published:** 2007-02-09

**Authors:** Dirk Walther, Roman Brunnemann, Joachim Selbig

**Affiliations:** 1 Max Planck Institute for Molecular Plant Physiology, Potsdam, Germany; 2 Institute for Biochemistry and Biology, Potsdam University, Potsdam, Germany; RIKEN Genomic Sciences Center, Japan

## Abstract

Regulation of gene expression via specific *cis*-regulatory promoter elements has evolved in cellular organisms as a major adaptive mechanism to respond to environmental change. Assuming a simple model of transcriptional regulation, genes that are differentially expressed in response to a large number of different external stimuli should harbor more distinct regulatory elements in their upstream regions than do genes that only respond to few environmental challenges. We tested this hypothesis in Arabidopsis thaliana using the compendium of gene expression profiling data available in AtGenExpress and known *cis*-element motifs mapped to upstream gene promoter regions and studied the relation of the observed breadth of differential gene expression response with several fundamental genome architectural properties. We observed highly significant positive correlations between the density of *cis*-elements in upstream regions and the number of conditions in which a gene was differentially regulated. The correlation was most pronounced in regions immediately upstream of the transcription start sites. Multistimuli response genes were observed to be associated with significantly longer upstream intergenic regions, retain more paralogs in the *Arabidopsis* genome, are shorter, have fewer introns, and are more likely to contain TATA-box motifs in their promoters. In abiotic stress time series data, multistimuli response genes were found to be overrepresented among early-responding genes. Genes involved in the regulation of transcription, stress response, and signaling processes were observed to possess the greatest regulatory capacity. Our results suggest that greater gene expression regulatory complexity appears to be encoded by an increased density of *cis*-regulatory elements and provide further evidence for an evolutionary adaptation of the regulatory code at the genomic layout level. Larger intergenic spaces preceding multistimuli response genes may have evolved to allow greater regulatory gene expression potential.

## Author Summary

The induction or repression of specific genes has evolved in living organisms as a mechanism to respond to environmental changes. At the molecular level, this process is mediated via molecular switches, so-called regulatory elements, generally located in the genomic region adjacent to the gene they control, the gene promoter. Upon environmental change, specific proteins bind to such regulatory elements, thereby turning on or off the associated genes. As this molecular response is often specific to the external signal, genes that respond to a large number of different external stimuli should harbor more distinct regulatory elements in their promoter regions than should genes responding only to few environmental challenges. In analyzing data for the plant *Arabidopsis thaliana,* we observed that indeed an increased number of regulatory elements is associated with a broader range of responses. Several other genome structural properties, such as gene size, the occurrence of similar genes in the *Arabidopsis* genome, and the distance between genes, were also observed to be correlated with a broader breadth of response. The results suggest that greater regulatory complexity appears encoded by an increased density of regulatory elements and provide further evidence for an evolutionary adaptation of the regulatory code at the genomic architectural level.

## Introduction

The regulation of gene expression has evolved in cellular organisms as a major adaptive mechanism to respond to environmental changes [1–5]. How the apparent diversity of responses is encoded in an organism's genome is a central question in understanding transcriptional regulation induced by different environmental and extracellular conditions [6–10]. The induction or repression of particular genes in response to specific environmental challenges is primarily controlled by the recognition and binding of transcriptional regulator proteins (transcription factors) to *cis*-regulatory elements constituted by short DNA sequence motif sites located in the upstream regions of genes [11–13]. Under the simplest scenario of transcriptional regulation, distinct external challenges are matched by specific cognate regulatory sites in upstream regulatory regions of genes that have evolved to respond to the particular perturbation. Genes that are differentially expressed in response to a large number of different external stimuli (multistimuli response genes) are therefore expected to contain more distinct *cis*-regulatory elements in their upstream regions than are genes that respond to only few environmental cues. There are two plausible strategies of how evolution may have shaped the noncoding, regulatory segments of genomes to encode a greater capacity of downstream genes to respond to a wider range of different stimuli by differential gene expression. A broader response spectrum may have evolved via an increased density of regulatory motifs or via an enlarged size of regulatory intergenic regions to accommodate more elements (or both). In analyzing expression patterns of Caenorhabibditis elegans and Drosophila melanogaster genes in different developmental and tissue differentiation stages, Nelson and coworkers [10] observed that indeed there exists a significant positive correlation between the complexity of a gene's expression, that is, to be expressed in a number of different tissues and developmental stages, and the size of its flanking noncoding, intergenic sequence, suggesting that regulatory requirements may have played a significant role in shaping the architecture of genomes. The association of promoters harboring multiple different regulatory sites with differential responses of their downstream genes to varied growth conditions has also been conceptualized by Harbison and coworkers using ChIP-chip yeast data [8]. They distinguished four types of motif arrangements in promoters: single regulators—associated with genes of common functions, repetitive motifs—allowing graded transcriptional responses, multiple regulators—allowing responses in diverse conditions, and co-occurring regulators—for physically interacting regulators.

In this study, we test and quantify the strength of the association of the presence of multiple different regulatory motifs in promoters with the breadth of differential gene expression response to external stimuli in Arabidopsis thaliana. For this purpose we use the available compendium of gene expression profiling data in the public AtGenExpress *Arabidopsis thaliana gene* expression repository (http://www.arabidopsis.org/info/expression/ATGenExpress.jsp) alongside previously characterized *cis*-element motifs mapped to upstream gene regions. *Arabidopsis* is an ideal model system for the investigation of the regulatory code in higher eukaryotic organisms due to its complete genome sequence [14], the availability of public domain resources of known *cis*-elements in upstream gene regions such as Athena (http://www.bioinformatics2.wsu.edu/cgi-bin/Athena/cgi/home.pl) [15], and genome-wide expression profiling data for a large and diverse collection of treatment-control experiments. These experiments encompass a wide range of abiotic and biotic treatments, the application of plant hormones and other chemical treatments performed, and were designed to enable comparative studies by using the same technology platform and reference conditions. We define the property of genes to be differentially regulated in response to many or few conditions as their “breadth of response.” It is this quantity and its relation to gene regulatory motifs and other genome structural properties that form the main focus of this study. Rather than internal gene expression regulation during tissue differentiation and organism development for sets of genes with available profiling data in developmental expression series and literature information as used by [10], the available *Arabidopsis* data allow us to assess systematically the differential gene expression response breadth and for nearly all genes more directly by measuring gene expression in response to external stimuli. Furthermore, with the mapping information of previously characterized regulatory motifs, albeit the set may likely not be considered complete, to the *Arabidopsis* genome at hand, we are able to associate gene expression complexity directly to *cis*-elements, their identity, frequency, and spatial distribution and in conjunction with genomic layout properties, such as distances between neighboring genes and gene size. Transcriptional response programs to external stress have been studied in microorganisms, yeast in particular [3]. Studying the association of regulatory capacity and the breadth of transcriptional response in a higher, multicellular and multitissue organism such as *Arabidopsis* will allow comparison and an assessment of the generality of the observed mechanisms.

Our results obtained in *Arabidopsis* lend further support to the notion that larger intergenic regions may have evolved to allow broader differential gene expression capacity [10]. *Arabidopsis* genes showing differential gene expression in response to a greater range of external stimuli are flanked by larger intergenic regions. In addition, increased breadth of gene expression response was observed to correlate with an increased density of motifs in upstream promoter regions, most pronounced in segments immediately upstream of the transcription start site. Among the various *cis*-elements analyzed, the TATA-box motif appears to play a unique role. We observed TATA-box–containing genes to possess a significantly increased breadth of response and to be associated with significantly longer upstream intergenic regions compared to TATA-less genes, thereby possibly allowing for greater regulatory capacity. Identified correlations of several fundamental genome architectural properties with the observed breadth of differential gene expression response are discussed in the context of evolutionary forces shaping the structure of eukaryotic genomes.

## Results

**Figure 1:**
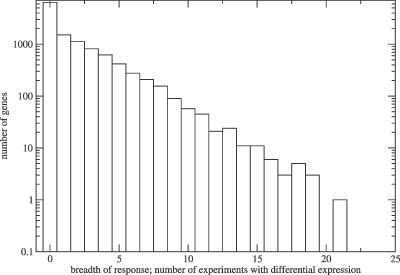
Semilogarithmic Frequency Distribution of the Number of Different Experiments in which Genes Were Found to Be Differentially Expressed Defined Here as the Breadth of Differential Gene Expression Response

Applying a noise-filtered threshold of 2-fold up-regulation or down-regulation in 43 treatment-control experiments, we observed a nearly exponential decrease in the number of genes with increasing cumulative numbers of experiments with differential expression (Figure 1). Most genes were found to be differentially expressed in only few experiments, whereas only a small number of genes were observed to respond to many different external stimuli.

Genes involved in stress response, cell growth, and lipid transport are particularly overrepresented in the set of multistimuli-sensitive genes (Table 1), whereas the housekeeping functions, protein catabolism and synthesis, RNA processing, and DNA repair, as well as uncharacterized genes with as-of-yet-unknown function, are more associated with the group of genes with narrow breadth of differential gene expression response, indicating that they are constitutively expressed. Genes involved in embryonic development were also grouped with narrow response genes. However, this may primarily be explained by the absence of embryonic development samples in the analysis. The gene *AtEXPA8* (At2g40610), a member of the α-expansin gene family and involved in cell wall modification and loosening, was observed to show the greatest response breadth and was differentially expressed in 22 different experiments.

**Table 1:**
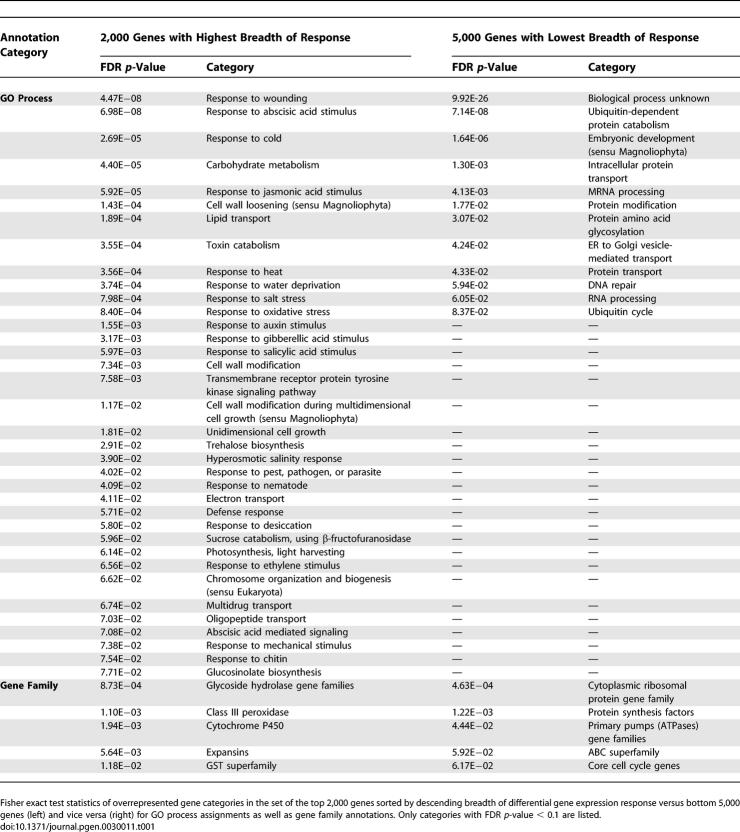
Classification of Genes with High or Low Breadth of Differential Gene Expression Response

**Figure 2:**
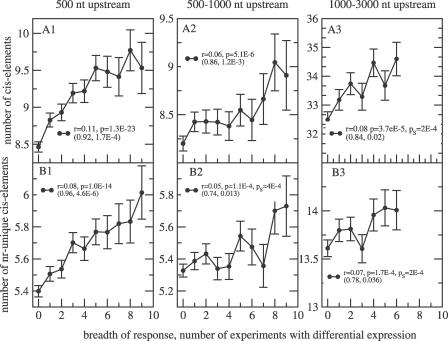
Correlation of the Number of *cis*-Regulatory Elements in Gene Upstream Promoter Regions of Different Length and Position Relative to the Transcription Start Site with the Breadth of Differential Gene Expression Response The upper row (A1–A3) shows data for all *cis*-elements including counts for multiple occurrences of *cis*-elements. In the lower row (B1–B3), only unique *cis*-element counts (see Methods) have been used. Genes were only included if their associated intergenic upstream region was large enough to fully contain the considered upstream interval, i.e., no overlap of preceding genes and the considered promoter segment was allowed. Shown are the mean values and the SEM. Correlation coefficients, *r*, and associated *p*-values are given for all raw data pairs (individual genes and associated motif counts) and, in parentheses, for the mean values as they are plotted in the graph. If the shuffled *p*-value, *p_S_*, was not zero, it is reported as well (see Methods). Mean *cis*-element counts were only plotted if at least 100 genes were detected at the particular breadth of response. Note: With increasing distance of the considered promoter region from the transcription start site, fewer genes were considered as intergenic upstream regions were frequently not large enough (respective gene counts were A1, B1 = 8,597; A2, B2 = 6,537; A3, B3 = 2,737).

With the mapping information for 93 previously characterized *cis*-element motifs to all *Arabidopsis* upstream gene regions up to a length of 3,000 nucleotides available from the Athena database [15] (see Methods), it is now possible to correlate the breadth of differential gene expression response to the number of *cis*-regulatory elements for all genes. If a broader differential gene expression response is reflected and even encoded by an increased number of *cis*-elements, we expect a positive correlation. Indeed, very significant positive correlations can be observed for both the number of total and unique elements in promoters of defined lengths (Figure 2), i.e., multistimuli response genes harbor more *cis*-elements in their upstream regions (higher density of elements) than do genes with a narrower scope of responses. This positive correlation was particularly significant and pronounced for an assumed promoter length of 500 nucleotides immediately upstream of the presumed site of transcription initiation. While the significance of the observed correlations was high, absolute motif counts increased only moderately corresponding to an increase of approximately 13% in the 500 nucleotides immediately upstream over the observed range of response breadth. For promoter segments farther upstream, the significance of the observed correlations was weaker, in part explained by fewer observations, albeit detectable, and the relative increase in motif counts smaller with increasing breadth of response.

The number of experiments with differential expression, the breadth of response, includes counts for both up-regulation and down-regulation in treatment-control samples. When only up-regulation or only down-regulation responses were considered differential gene expression events, we observed that the obtained positive correlations of motif counts with breadth of response was primarily caused by up-regulation rather than by down-regulation events for which no significant correlation were obtained to the number of upstream motifs in promoter segments of defined length (Figure S2).

**Figure 3:**
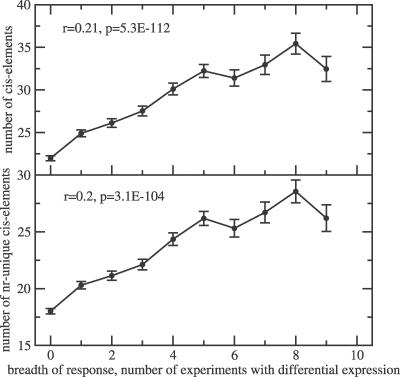
Correlation of the Number of Regulatory *cis*-Elements in Gene Upstream Promoter Regions Up to 3,000 Nucleotides in Length or Truncated at the Next Upstream Gene Boundaries (Variable Promoter Length) with the Breadth of Differential Gene Expression Response Plotted are the mean values and associated standard errors of the mean. Pearson linear correlation coefficients, *r,* and associated *p*-values are given for all raw data pairs (for all genes considered in the analysis).

Determining the correct length of gene promoters, i.e., to assess whether a putative *cis*-acting regulatory site will exert an influence on its downstream gene depending upon its distance from the site of transcription initiation, is difficult experimentally, even more so if only *in silico* mapping information is available. Allowing variable promoter segment lengths of up to 3,000 nucleotides, but excluding *cis*-elements that overlap with neighboring upstream genes and only considering *cis*-elements that map to intergenic upstream regions, the magnitude of the correlation of motif counts to breadth of response increased significantly (*r* ≈ 0.2 obtained for motif counts in variable-length promoter segments [Figure 3] compared to *r* ≈ 0.1 for fixed promoter lengths [Figure 2]). Genes that are differentially regulated in nine different experiments (the greatest observed breadth of response value in the set of experiments analyzed here with more than associated 100 genes) have 50% more *cis*-elements for both overall *cis*-element counts and unique counts versus genes with no detectable differential response in the experiments included in this study.

**Figure 4:**
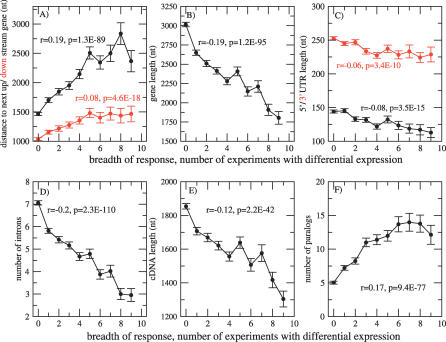
Correlation of the Length of Intergenic Upstream and Downstream Sequence (Distance to the Next 5′-Upstream Gene), Gene Length, 5′/3′ UTR Length, Number of Introns, cDNA Length, and Number of Paralogs with the Breadth of Differential Gene Expression Response (A) Intergenic upstream and downstream sequence (distance to the next 5′-upstream gene). (B) Gene length. (C) 5′/3′ UTR length. (D) Number of introns. (E) cDNA length. (F) Number of paralogs with the breadth of differential gene expression response. Plotted data points correspond to mean values and associated standard errors of the mean. Pearson linear correlation coefficients, *r*, and associated *p*-values are given for all raw data pairs.

This result suggests that the length of the upstream intergenic region, too, is positively correlated with the breadth of differential gene expression response. Such a positive correlation has already been reported in analyses of gene expression profiles in different developmental and cell differentiation stages in C. elegans and D. melanogaster [10]. Indeed, we found a very significant positive correlation of intergenic upstream sequence length and the breadth of response (*r* = 0.19, *p* = 1.2E−89; Figure 4A) also in response to external stimuli. On average, highly multistimuli response genes have approximately double the intergenic upstream space compared to genes with no differential response. This trend was observed equally strong for both differential up-regulation or down-regulation events (Figure S3). This finding prompted us to relate other gene properties to the breadth of response. The length of downstream intergenic segments was also found positively correlated with breadth of response, albeit at a less significant level and smaller magnitude (*r* = 0.08, *p* = 4.6E−18; Figure 4A) than for the corresponding upstream segment lengths. Downstream intergenic segments are also significantly shorter than upstream segments. We observed that multistimuli response genes are, on average, significantly shorter, with shorter gene length (*r* = −0.19, *p* = 1.2E−95 [Figure 4B]) as well as shorter cDNA length (*r* = −0.12, *p* = 2.2E−42 [Figure 4E]) and have shorter 5′ (*r* = −0.08, *p* = 5.8E−16) and, to a lesser degree, 3′ untranslated regions (*r* = −0.05, *p* = 5.1E−9 [Figure 4C]). Commensurate with shorter gene length, the number of introns was also observed to be negatively correlated with breadth of differential expression (*r* = −0.2, *p* = 2.3E−110 [Figure 4D]), as was the mean length of intronic segments (*r* = −0.2, *p* = 6E−114). In addition to the analyzed gene size–related parameters, multistimuli response genes are also more likely to contain additional paralogs in the *Arabidopsis* genome than are narrow-response genes (*r* = 0.17, *p* = 9.4E−77 [Figure 4F] [30% amino acid sequence identity], and *r* = 0.09, *p* = 3.1E−24 for the more stringent paralog settings of requiring 70% amino acid sequence identity [see Methods]). Among the various properties analyzed, the length of the upstream region and gene length and the associated number of introns were found most strongly correlated with breadth of differential gene expression response. The sum of the intergenic upstream and downstream distances measuring the size of a gene's intergenic flanking region was not observed to be negatively correlated with the size of the flanked gene (*r* = 0.014, *p* = 0.14); i.e., genes are not distributed evenly such that longer intergenic regions follow from shorter genes. Applying a multiple linear regression approach, we analyzed what level of correlation with breadth of response can be achieved by combining the various and largely independent properties depicted in Figure 4. Performing a stepwise-forward multiple linear regression and using nonunique motif counts in the 500-nucleotide upstream regions (motif density), the three most significant regressors were (1) number of introns (partial correlation coefficient, β = −0.23), (2) distance to next upstream gene (β = 0.13), and (3) motif density (β = 0.09), yielding a combined level of correlation of *r* = 0.28, *r* ^2^ = 0.08 (*p* ≪ 0.01, *n* = 6,976 genes with complete information and upstream intergenic region longer than 500 nucleotides). Adding more properties (downstream region length, UTR lengths, etc.) did not result in significantly increased correlation levels.

We investigated what gene families and functions are associated with long upstream intergenic segments. By sorting all *Arabidopsis* genes according to their upstream intergenic length and comparing the Gene Ontology (GO) annotations of a subset of genes with longest upstream segments to a set of genes with shortest upstream segment lengths using Fisher exact statistical tests, we found that transcription factors, transcriptional regulatory functions, and genes involved in signaling processes are particularly overrepresented among genes with long intergenic upstream segments, while ribosomal genes involved in protein biosynthesis and genes involved in other housekeeping functions such as glycolysis are relatively overrepresented among genes with short upstream segment lengths, as are genes with currently unidentified function (Table 2). The found association of functional categories with intergenic upstream distances in A. thaliana agrees well with very similar observations reported for C. elegans and D. melanogaster [10].

**Table 2:**
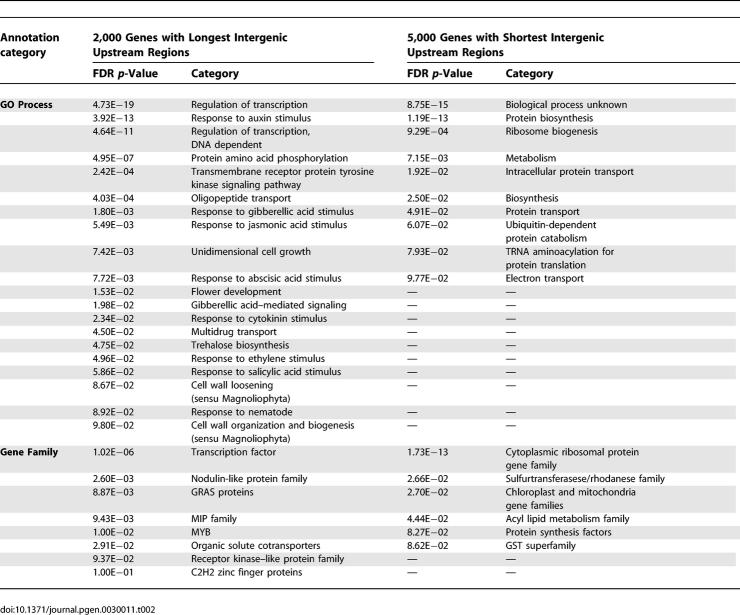
GO Process and Gene Family Annotations Overrepresented in Genes with Long (Left) and Short (Right) Intergenic Distances to Next Upstream Gene (FDR *p*-Value < 0.1)

We analyzed which gene families and biological processes are associated with genes with the greater number of *cis*-elements in their upstream promoter region. When counting regulatory elements in upstream segments of up to 3,000 nucleotides and truncating them at neighboring upstream genes, i.e., variable promoter length, the GO processes and gene families characteristic for genes with many motifs largely coincided with the profile obtained for genes with long upstream segments (Table 2). Likewise, profiles for genes with few motifs matched profiles obtained for genes with short upstream regions. As the motif density was observed to be relatively constant across intergenic regions (Figure S4), this agreement is not surprising because motif counts are positively correlated with intergenic distances. When we confined the promoter count to upstream regions of 500 nucleotides, i.e., analyzing the density of motifs in 500 nucleotides, only one GO process (response to wounding) was borderline significant (false discovery rate [FDR] *p*-value = 0.09). No other GO process category or gene family association was found to be significantly correlated with either high or low motif density (FDR *p*-value > 0.1 for all categories and gene families). However, “response to wounding” and “response to cold” were ranked highest among GO processes associated with high motif density. Several other response-related categories were also contained among the top-ranked processes, such as “response to oxidative stress” (rank 6) and “response to gibberellic acid stimulus” (rank 16) and, as a single-word category, “response” was very significantly associated with high motif density (FDR *p*-value = 5.1E−7), suggesting that higher motif densities are indeed associated with genes that are involved in environmental response pathways.

Well-known stress response elements in plants such as the ABRE-like, ABF, and DRE binding site motifs [16] are among the motifs found most frequently in genes with large breadth of response (Table 3). The occurrence of the TATA-box motif, a commonly found eukaryotic core promoter element involved in transcription initiation and usually located approximately 25 to 30 nucleotides upstream of the transcription start site [17], also appears to be preferentially associated with multistimuli response genes. Genes with a putative TATA-box motif present in the 60 nucleotides upstream of the transcription start site had a significantly greater breadth of response (mean number of experiments with differential expression = 3.4, *n* = 2,002) than did genes lacking a TATA-box motif in their 60-nucleotide upstream region (mean number of experiments with differential expression = 1.9, *n* = 9,795, *p* = 5.0E−113). Consistent with increased intergenic upstream distances for multistimuli response genes, TATA box–containing genes were also observed to have longer upstream regions (2,370 nucleotides versus 1,737 nucleotides for TATA-less genes, *p* = 3.4E−34). Motifs reported to control housekeeping genes (TELO-box promoter motif [18]; hexamer promoter motif [19]) or implicated to confer tissue-specific expression (LEAFYATAG [20]) were found to be more associated with genes with narrow range of differential expression response, i.e., their expression is constitutive or their differential expression response is very specific.

**Table 3:**
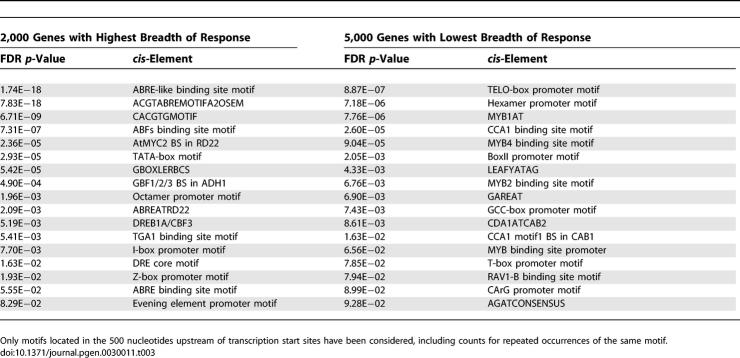
*cis*-Regulatory Elements (Motifs) Associated with Genes with High (Left) versus Low (Right) Breadth of Differential Gene Response and vice versa at Significance Level of FDR *p-*Value < 0.1

It is conceivable that in transcriptional regulatory signaling cascades, early responders evolved a greater sensory capacity (i.e., respond to many different transcription factors) and then channel the response to common effector genes. In our framework, equating sensory potential to the number of *cis*-elements, we then expect early responders to be associated with a greater number of regulatory elements. The 18 AtGenExpress abiotic stress time series datasets (nine for root and shoot tissue, respectively) allowed testing of this hypothesis. Comparing genes that are differentially expressed during the first 3 h after stimulus to genes that respond at later time points and assuming a fixed promoter length of 500 nucleotides, early-response genes were associated with only marginally or no increased motif count densities with 3% (2%) more (nonredundant [nr]-unique) motifs in 500 nucleotide–upstream regions compared to late genes (7.5 versus 7.3, *p* = 5.9E−12; 5.8 versus 5.7, *p* = 3.4E−6 for nr-unique motifs, respectively). However, in allowing promoter lengths of up to 3,000 nucleotides and truncating them at neighboring upstream gene sites (variable maximal promoter lengths), early genes were observed to harbor 20% (16%) more (nr-unique) motifs than late genes (34.4 versus 28.5, *p* = 2.1E−170; and 13.2 versus 11.4, *p* = 1.2E−179 for nr-unique motifs, respectively). As was observed before, an increased number of *cis*-elements for a specific group of genes may originate from two sources: higher motif density and more different motifs and, apparently more significantly, longer upstream regions providing more space for potential regulatory sites. Early-response genes have, on average, 30% longer intergenic upstream segments than do late-response genes (2,668 nucleotides versus 2,064 nucleotides, *p* = 2.9E−115) and are more likely to contain TATA-boxes (25% versus 19%, Fisher exact *p*-value = 1.4E−12). Consistent with a transcriptional regulatory signaling cascade, genes involved in the regulation of transcription, signaling, as well as stress response genes are overrepresented among early-responder genes, whereas ribosomal genes and genes generally involved in protein biosynthesis, metabolism, and other nonsignaling processes are more characteristic of late-response genes (Table 4).

**Table 4:**
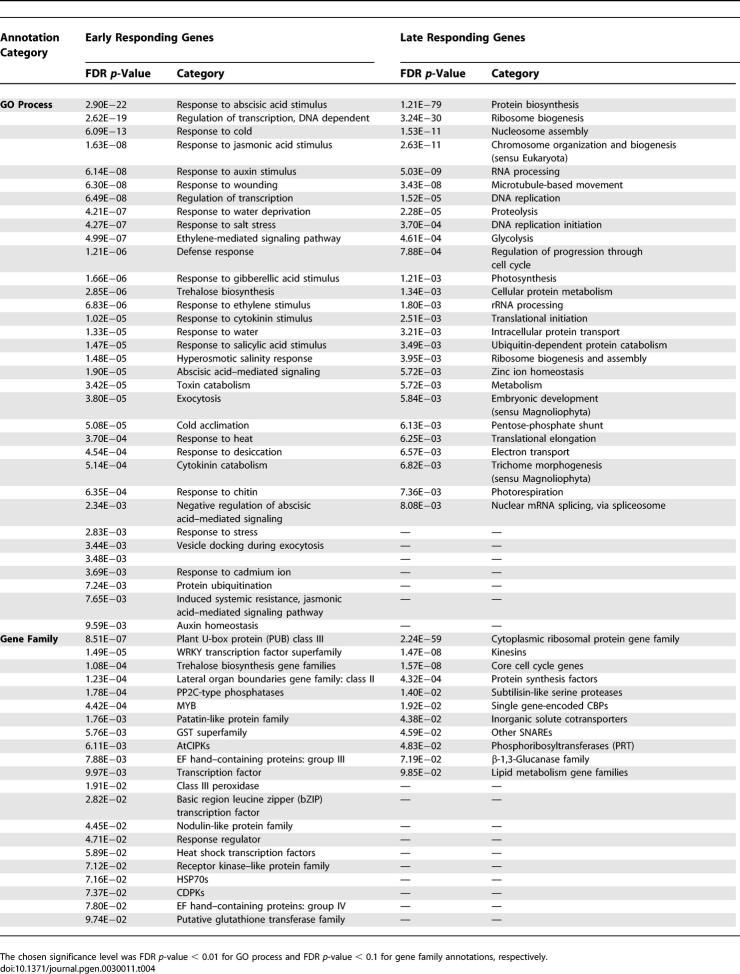
GO Process and Gene Family Annotations Associated with Genes Differentially Expressed Early (3 h or Earlier [Left]) and Late (After 3 h [Right]) after Abiotic Stress Stimulus in Nine different Abiotic Stress Time Series Experiments and in Root and Shoot Tissue, Respectively (Table S1)

## Discussion

The objective of our investigation was to test whether there is a significant relationship between encoded, by way of *cis*-regulatory motifs, and actually observed differential gene expression diversity. Our primary focus has been the relationship of *cis*-regulatory motif counts and differential gene expression using data from A. thaliana. In discussing our results, we will first consider several technical aspects of this study and then turn to the biological relevance of our findings.

The currently available data for both data types, *cis*-element motifs and differential gene expression, are by their very nature associated with high levels of noise. The set of *cis*-elements for every gene was obtained by computational mapping of short sequence motifs to upstream gene promoter regions. Without experimental verification, a large number of such predicted sites are expected to be false positives as it is difficult to determine how far upstream the promoter region actually extends, with the exceptions that promoters may not overlap with neighboring gene segments, and where the boundaries of the core-promoters—the site of constitutive transcription initiation with less differential regulatory function—are located. Furthermore, alternative transcription start sites have been reported for up to 50% of the *Arabidopsis* genes [21], associated perhaps with separate regulatory regions. There are also likely to be more, as of yet unknown, motifs than the 93 that were considered here, as there are about 1,500 transcription factors encoded in the *Arabidopsis* genome [22].

On the expression side, detecting true differential expression with only few technical and biological replicate samples is difficult. We applied a simple criterion for calling differential expression as the objective has not been to identify high-confidence gene expression markers for the various conditions but rather to detect global quantitative trends.

However, the fact that we still observed significant correlations between both quantities, despite the high noise levels, suggests that the true correlation between both quantities may, in fact, be even more significant and of greater magnitude than estimated in this study. This is particularly relevant for the investigated correlations between density of motifs and breadth of response (Figure 2).

On the other hand, high noise levels increase the risk of artefacts. One such source of artificial distortion of the data may be the normalization of raw gene expression values. To test the robustness of the observed correlations of motif counts to the breadth of differential gene expression response, we applied two additional normalization techniques (MAS 5.0; Affymetrix proprietary software, http://www.affymetrix.com) and GCRMA (Affymetrix) [23] and compared the results to the data obtained from using Robust Microchip Analysis (RMA; Affymetrix) [24]. Almost identical trends of motif counts in the 500-nucleotide gene upstream regions to the breadth of response have been obtained for all three normalization methods (Figure S5).

In limiting the analysis to genes with expression levels for which no dependence of the breadth of differential response on the expression level was noticeable (Figure S1), we applied additional caution to guard against artefacts. However, the primary observations and conclusions presented here also hold when all genes, including the 10,000 genes with low expression levels in control samples, are used in the analyses.

We have counted *cis*-elements in upstream region as they are presented in the Athena resource regardless of their mapping orientation (forward or reverse strand). Therefore, we compared results from using only motifs that map in the same orientation as the coding strand for a given transcript and obtained qualitatively identical results. Evidently, absolute counts were lower. In addition, there is evidence that *cis*-elements can be recognized in both orientations (bidirectional motifs). For example, correlated expression of genes transcribed in opposite direction that are in close proximity to one another suggests that *cis*-elements are shared between the two genes regardless of direction [25].

### Regulatory Capacity Is Encoded by Both Motif Density and Absolute Motif Counts Correlated to Intergenic Upstream Space

In this study, we followed the notion that regulatory capacity is associated with information-bearing properties of the intergenic region upstream of the transcription start site of the regulated genes. The simplest and most accessible measure of information content was to correlate *cis*-regulatory motif counts to the breadth of differential gene expression response. We found that increased breadth of response is indeed positively correlated with greater motif density (Figure 2). While the observed correlations were highly significant, their magnitude was generally low. The positive correlation between breadth of response and motif count was relatively strongest for the first 500 nucleotides upstream compared to the other investigated regions, suggesting that this interval may generally be the most relevant promoter segment to control gene expression. Apart from greater density, more motifs and generally greater information content can also arise from more available intergenic space. Analyzing gene expression profiles in different developmental and cell differentiation stages in C. elegans and *D. melanogaster,* Nelson and coworkers [10] observed that genes with more complex expression profiles were indeed associated with larger flanking intergenic intervals. Genes with regulatory functions were shown to generally have longer upstream regions, whereas genes involved in housekeeping functions have shorter upstream segments. The results reported here for *Arabidopsis* and analyzing differential expression data for transcriptional response to external stimuli confirm these findings. The requirements for encoding regulatory response complexity to external environmental challenges also appear to have played a role in shaping the layout of the *Arabidopsis* genome. Not conflicting with this result, we saw that motif density also is varied in correlation with different complexity of transcriptional response (Figure 2), suggesting that both principal mechanisms (greater density and/or more space) were employed by evolution to encode complex transcriptional response patterns. While both flanking sequences (upstream and downstream) showed positive correlation of their length to the breadth of response, we found that in *Arabidopsis,* upstream distances were more strongly correlated with response diversity and were generally longer (Figure 4A) than downstream intervals, suggesting that the information content upstream of a gene may be more relevant to the encoding of regulatory properties than downstream segments. Apart from transcription factor binding sites, other mechanisms and motifs, such as histone binding sites, chromatin structural changes, enhancers, silencers, and insulators, as well as sites of epigenetic regulation, may also constitute regulatory elements contained in intergenic segments that can contribute to more complex regulatory properties encoded in longer upstream intervals.

Alternative to our assumption that increased breadth of response correlates with increased regulatory capacity, it might be speculated that because genes that respond to many different stimuli in a similar manner, they are regulated by a common regulatory mechanism and therefore should be expected to be regulated by fewer rather than more *cis*-elements. However, data presented by Gasch et al. [3] on the gene expression response to environmental stress in yeast and the results presented here indicate that multistress response genes appear to be controlled by different and condition-specific regulatory mechanisms.

It has been recognized that often multiple factors are involved in the initiation of transcription, suggesting a combinatorial control of transcription initiation [26,27]. A combinatorial use of the repertoire of *cis*-regulatory elements enlarges the regulatory coding capacity tremendously. Conceptually, the basic premise of this investigation—a positive correlation between encoded and observed differential gene response—also applies in the case of combinatorial control.

### Multistimuli Response Genes Are Shorter in *Arabidopsis*

The correlation between expression level and gene size and associated properties such as number and length of introns has been investigated in several studies [28–34]. Surprisingly, while highly expressed genes were found to be shorter in animals and to harbor fewer and shorter introns [30], they appear least compact and relatively largest in plants [33] and C. elegans [29]. Several evolutionary scenarios such as selection for economy and genomic design have been discussed to explain the observed trends [31,34]. Using the AtGenExpress dataset analyzed in this study, we also observed a general increase in gene size, intron length, and intron number with expression level in *Arabidopsis*, albeit for very highly expressed genes, the trend was reversed (Figure S7).

The focus of this study has been on the correlation between breadth of differential genes expression response, not its absolute level, and genome architectural properties. It can be assumed that differential gene expression is largely independent of expression level with the obvious boundary effects that lowly expressed genes are more likely to be up-regulated and highly expressed genes have a greater chance of being down-regulated as certain expression levels cannot be exceeded. For medium expression levels, indeed no such correlation was found in our dataset and deviations at low levels likely caused by noise (Figure S1).

Interestingly, we observed that properties related to gene size (gene length, cDNA length, number of introns) are strongly and negatively correlated with breadth of differential gene expression response (Figure 4). While this may reflect a coincidence that informative (involved in regulation or signaling) gene families are generally smaller, the intriguing question arises of whether transcriptional efficiency may have played a role during evolution. Shorter genes with fewer introns may be produced more economically and thereby quicker in response to sudden external stimuli. In fact, the number of introns was the strongest, albeit marginally, of all properties analyzed to correlate with breadth of response (Figure 4 and multiple linear regression results). Informative molecules may also be smaller as they need to diffuse within the cell or tissues to carry their information to the place of perception rather than being constituents of larger and more static cellular machineries. Clearly, comparing our findings in *Arabidopsis* with those in warm-blooded animals will shed further light on the generality of our observations.

### Special Role of TATA-Boxes

Among the *cis*-regulatory motifs considered in the analysis, the preferential association of the TATA-box motif—a commonly found eukaryotic core promoter element involved in transcription initiation and usually located approximately 25 to 30 nucleotides upstream of the transcription start site [17]—with multistimuli response genes is of particular interest. In the gene set studied here, 17% of all genes contained TATA-box motifs within the first 60 nucleotides upstream of the transcription start. TATA-box genes were found associated with stress response and were shown to be subject to chromatin remodeling factors consistent with their regulation by nucleosomal mechanisms [35]. The TATA-box motif was also described recently to confer increased interspecies gene expression variation of the corresponding genes [36]. Our observation that TATA-box–containing genes have longer intergenic upstream regions is consistent with their chromatin and nucleosomal regulation and also suggests that the expression of TATA-box genes may evolve at higher rates, causing increased variation across species because their upstream regulatory potential is greater and, therefore, more amenable to change and modulation. Interestingly, presence of the TATA-box also strongly correlates with a decreased number of introns in the downstream gene (number of introns for genes with TATA: 3.7, without: 5.9; *p* = 1.9E−93). It is presently not clear whether this correlation indicates a direct coupling between transcription and splicing [37] or whether it originates from an indirect correlation via a common principle shaping the genome, such as possibly the breadth of response and the role of both properties in defining it as reported here.

### Multistimuli Response Genes Have More Paralogs

Assuming that multistimuli response or “informational hub” genes play more critical roles than genes that have a narrower response scope, it can be speculated that failures of such central genes—by spontaneous disruptive mutations, for example—may be more detrimental to the organism. Therefore, functional backup genes may have evolved to safeguard against such failures. A plausible evolutionary solution would be to retain copies or paralogs of hub gene in the genome from gene or segmental duplication events, i.e., genes with identical or similar function [38,39]. The observed increase of the number of paralogs with increasing breadth of response is consistent with the concept of selection for functional backup (Figure 4F). However, results reported in yeast suggest that rather than redundancy provided by duplicated genes, interactions between unrelated genes appear to be responsible for robustness against mutations [40]. Furthermore, rather than a static robustness provided by “replacement parts,” a dynamic reprogramming of the transcriptional regulatory network may be employed during “fail-safe” scenarios [41].

We saw that multistimuli response genes were generally associated with environmental response processes (Table 1). As the number of different external stimuli is large and fine-tuning the perception as well as signal transduction depending upon the stimulus will likely be beneficial, it may have been evolutionarily advantageous to use duplication events to evolve genes (paralogs) with shifted and novel response scope, also explaining a greater number of paralogs for multistimuli response genes [42].

Alternatively, paralogs may allow a greater dynamic range of the response to external stimuli. Corresponding dosage effects and their role in the retention of paralogs have recently been discussed in the literature [43].

### Stress Response Genes Have Greater Breadth of Differential Response—A Potential Experiment Bias

We observed that genes implicated in the response to specific stresses (e.g., cold) are also among the genes with a very broad range of differential gene expression in response to various environmental changes (Table 1). However, some of the applied external changes correspond to very similar environmental cues (salt and osmotic stress, for example) and common transcriptional response programs will likely be triggered. Ideally, only very different and unrelated external stimuli would be used in our analyses, but imposing such requirements would reduce the number of experiments to very low numbers. Therefore, it needs to be borne in mind that the similarity between different external conditions and the resulting relative overrepresentation of particular types of external stimuli may cause a bias toward certain gene functional categories (salt stress response, for example). Interestingly, when the abiotic stress series was excluded from the list of experiments (about half of all experiments, Table S1), general stress response GO categories, including abiotic stress response, were still overrepresented among the genes with high breadth of response (“response to wounding,” FDR *p*-value = 3.7E−7; “response to oxidative stress,” FDR *p*-value = 5.5E−4; “response to heat,” FDR *p*-value = 0.04), suggesting that some stress response genes may truly display a large and diverse range of differential response.

### Characteristic Sequence Signatures in Longer Upstream Regions of Multistimuli Response Genes?

If, as observed, longer upstream regions are associated with greater gene expression complexity of downstream genes, the question emerges of whether there are characteristic sequence motifs in longer regions that are not found in short intergenic upstream segments. Such motifs may help identify and elucidate new regulatory elements and even mechanisms, DNA structural properties, and their influence on gene regulation, for example. Recent reports that large fractions of the nontranslated segments of genomes are functionally important based on analyses of mutation rates [44] and the greater-than-expected occurrences of specific sequence patterns in noncoding DNA segments associated with genes involved in signaling and transcription regulation processes [45] strongly encourage the pursuit of further research in this direction.

As sessile plants cannot evade changing and adverse environments, they may rely more strongly on elaborate transcriptional response programs and may thus serve as ideal model systems for the study of the regulatory code in the genomes of higher organisms.

## Materials and Methods

### Gene expression data.

Gene expression information was obtained from AtGenExpress. Profiling data based on the ATH1 Affymetrix GeneChip microarray platform [46] from the abiotic stress, biotic, nutrient, hormone, and chemical treatment-control series have been used. A detailed list of the 43 experiments, included samples, and a brief description of the various conditions, is available in Table S1.

### Gene expression data normalization.

Raw expression data files were obtained for treatment and associated control samples. All samples associated with a particular treatment-control experiment were preprocessed and normalized together using the Affy-package [47] available in the Bioconductor software suite [48]. Raw expression level data were normalized applying three different methods: RMA [24], GCRMA [23], and MAS 5.0 (Affymetrix). Unless noted otherwise, results are based on RMA normalization as the default normalization method.

### Differential expression criteria and probe selection, breadth of response.

Genes were considered differentially expressed if |m_T_ − m_C_| > 1 and s > 0.5 where s = |m_T_ − m_C_|/(σ_T_ + σ_C_) and m_T_, m_C_ denote the mean logarithm-base-2 transformed expression levels for associated gene probes across the available treatment (t) and control (c) replicate samples, and σ_T_, σ_C_ are the associated standard deviations of the log-2 expression levels. The first condition corresponds to a threshold of minimally 2-fold up-regulation/down-regulation of treatment expression levels relative to the levels in the associated control samples. The second criterion was introduced as a simple statistical significance measure similar to a simplified *t*-test. By normalizing to the standard deviation, rather than the standard error as done in the *t*-test, the risk of biasing the results to experiments with more repeats was reduced. The test metric s was introduced by Golub et al. [49] in their seminal study on cancer classification based on gene expression. To test whether the results are robust with regard to the chosen threshold values, other, more stringent, parameter values were applied. Qualitatively similar results were obtained (Figure S6). All mitochondrial and chloroplastidial gene probes were discarded. Only probes that map uniquely to annotated genes and with available *cis*-regulatory motif information have been considered. To ensure independence of the frequency of detected differential expression events on the absolute expression level and to avoid possible normalization artefacts, we examined for all gene probes the number of experiments in which a gene probe was observed to be differentially expressed as a function of the median rank of this probe across all control samples in the dataset and compared the results for all three normalization methods (Figure S1). For probe ranks greater than 10,000, no significant influence of the absolute expression level on the frequency of differential expression was observed. Therefore, we discarded the 10,000 probes with lowest median rank across all control samples from the analysis. Analyses using all probes have also been conducted and confirm the presented results. Applying the above criteria, 11,797 *Arabidopsis* genes were used in the expression data analysis using their unambiguously mapping array probes, i.e., every probe mapped to only one gene and every gene mapped to only one probe. Mapping information was obtained from The *Arabidopsis* Information Resource (TAIR) [50] and the ATH1-chip annotation file provided by Affymetrix. We define the term “breadth of response” for every gene as the cumulative number of treatment-control experiments in which the gene was found to be differentially expressed.

### *cis*-Regulatory elements.

Transcription factor binding site location information was obtained from the Athena promoter annotation resource [15]. In Athena, transcription factor site coordinates are obtained by sequence mapping of consensus motif sequences imported from the Plant *cis-*acting regulatory DNA elements (PLACE) [51] and *Arabidopsis* Gene Regulatory Information Server (AGRIS) [52]. The dataset contained mapping information for 93 different and previously characterized binding site motifs in the 5′ upstream gene segments of up to 3,000 nucleotides in length associated with genes and corresponding probes present on the ATH1 microarray platform.

### Construction of sets of an nr-unique *cis*-regulatory elements.

Mapping the 93 Athena motifs to gene promoter regions results in several redundancies and motif overlaps that were eliminated in order to construct truly unique sets of motifs and corresponding motif counts associated with every gene. Motifs were sorted alphabetically for every gene and assessed for uniqueness in consecutive order. Motifs whose mapping location either fully or only partially overlaps with already accepted motifs were not included in the unique set of motifs. Thus, redundancy was eliminated based on motif sequence information. The obtained set of unique *cis*-elements comprised a total of 76 different motifs, i.e., 17 motifs were excluded as they always overlapped with other motifs. For example, the motif DREB1A/CBF3 (consensus sequence “RCCGACNT”) contains the DRE core motif (consensus sequence “RCCGAC”). Thus, the DREB1A/CBF3 element was never included as a separate motif in the unique set as it always contains the DRE core motif which is sorted alphabetically before it. Multiple occurrences of the same motif at different locations in gene promoters were collapsed to only single counts in the nr-unique *cis*-element set.

### Correlation analysis and significance levels.

The strength and magnitude of the association of the various investigated gene properties to the number of experiments in which gene probes were observed differentially regulated (breadth of response) was quantified using linear regression and the linear Pearson correlation coefficient, *r*. Two types of correlation statistics have generally been computed: (1) the correlation of all value pairs for all probes and (2) for mean values only, for example, the mean number of *cis-*regulatory elements for genes differentially expressed in *n* different experiments. A minimum of at least 100 probes was required for mean values to be included in the latter, thus excluding mean values of lesser confidence. The significance of the correlation was assessed using standard *p*-value calculation for correlation coefficients based on the *t*-statistic with *t* = *r* × sqrt[(*N* − 2)/(1 − *r* ^2^)], and corresponding two-tailed *p*-values were computed from the *t*-distribution where *N* is the number of value pairs to be correlated. In addition to this parametric significance testing, a nonparametric method based on data shuffling was implemented. The pairing of data from two data vectors that were to be tested for correlation was randomly shuffled 10,000 times; i.e., one vector was repeatedly randomly shuffled. The count, *C*_S_, how often the magnitude of the correlation coefficient exceeded the actually observed coefficient for the unshuffled data, divided by the total number of shufflings, *N*_S_, served as a nonparametric *p*-value estimate, *p*_S_, for the correlation coefficients such that *p*_S_ = *C*_S_/*N*_S_. In almost all cases, the obtained *p*_S_-value was zero, i.e., no shuffled correlation coefficient was obtained with greater correlation than the actually observed one. We therefore only list *p*_S_-values in the few cases with nonzero values (Figure 2). All *p*-values reported for *t*-test comparisons of mean values correspond to the two-tailed value.

### GO data and categorical correlations by Fisher exact tests.

GO information was obtained from TAIR [50]. Association tests of categorical gene classification data (GO annotations or *cis*-regulatory elements) with two gene sets were performed using the Fisher exact test. The two one-tailed Fisher exact *p*-values corresponding to overrepresentation or underrepresentation of categories in the two sets relative to one another have been calculated based on counts in 2 × 2 contingency tables. Counts *n*_11_, *n*_12_, *n*_21_, and *n*_22_ in the contingency table refer to *n*_11_, number of observations of a particular category in the first gene set; *n*_12_, number of other categories in the first gene set; *n*_21_, number of observations of category in second gene set; and *n*_22_, number of observations of other categories in the second gene set. Listed *p*-values correspond to multiple testing-corrected Fisher exact *p*-values using the FDR method ([53]).

### Gene and genomic sequence and gene mapping information.

Gene and genomic sequence information and gene mapping information to the *Arabidopsis* genome sequence, including intergenic distances, number of introns, gene, cDNA, and 5′/3′ UTR length information, and all sequence information, were obtained from TAIR database release 6 [50]. For 2,238 of the 11,797 genes, more than one transcript sequence was contained in the TAIR dataset (annotated splice variants). For those genes, always the first instance was taken (“.1”-transcript) for the study of transcript-related properties such as UTR length or cDNA length. We verified that similar results were obtained when taking different instances and that the number of splice variants per gene was not correlated with the breadth of response. It should be borne in mind that the ATH1 probes were designed to target the 3′ end of gene transcripts [46] and, therefore, identification of various splice forms of genes is difficult or impossible.

### Identification of gene parologs.

Duplicated genes within the *Arabidopsis* genome were identified following a method introduced by Gu and coworkers [54,55]. This method is based on an all-against-all sequence comparison of protein sequences that are not splice variants of the same gene and require at least 30% amino acid sequence identity with adjusted higher thresholds for short sequences. A second grouping of genes into paralogous gene families was generated applying a more stringent threshold of 70% amino acid sequence identity.

### Multiple linear regression.

Multiple linear regression was performed using Statistica 7.1 (StatSoft, http://www.statsoft.com). Forward stepwise regression was applied with F to enter set to 1 and casewise missing value deletion.

## Supporting Information

Figure S1 Relationship between Number of Experiments (NE) in Which Genes Were Differentially Regulated as a Function of Absolute Expression Level Measured by the Median Rank of Gene Expression Levels Across All Control Experiments Curves correspond to the 500 point-running averages for the *x*-axis sorted data for the three different applied expression data normalization techniques.
(847 KB EPS)

Figure S2 Correlation of the Number of *cis*-Regulatory Elements in 500-Nucleotide Upstream Promoter Regions of Varying Length with the Breadth of Differential Gene Expression Response Red data points refer to counts of *cis*-elements in differentially up-regulated genes, whereas the green data points show data for down-regulated genes in treatment samples versus the respective control samples. Only genes with longer than 500-nucleotide intergenic upstream regions have been considered. Shown are the mean values and the standard errors of the mean (SEM). The *p*-values associated with the observed linear Pearson correlation coefficient are given for all raw data pairs (all gene probes and associated gene properties) and, in parentheses, for the mean values as they are plotted in the graph. Only mean values with more than 100 raw observations (genes) are plotted.
(16 KB EPS)

Figure S3 Correlation of the Length of the Intergenic Space in Nucleotides Preceding Genes with Their Breadth of Differential Gene Expression Response Red data points refer to differentially up-regulated genes, whereas the green data points show data for down-regulated genes in treatment samples versus the respective control samples. Shown are the mean values and the SEM. The *p*-values associated with the observed linear Pearson correlation coefficient are given for all raw data pairs (all gene probes and associated gene properties) and, in parentheses, for the mean values as they are plotted in the graph. Only mean values with more than 100 raw observations (genes) are plotted.
(15 KB EPS)

Figure S4 Motif Distribution in Upstream Intergenic Regions without Any Gene Overlaps (Green Histogram), with Possible Preceding Genes Falling into the 3,000-Nucleotide Intergenic Upstream Region Regulatory motifs are less frequent in *intra*genic regions.
(16 KB EPS)

Figure S5 Mean Number of *cis*-Elements in 500-Nucleotide Gene Upstream Regions and Associated Standard Error of the Mean as a Function of Observed Breadth of Differential Gene Expression Response for Three Different Expression Data Normalization Techniques as Indicated in the Figure Legend The correlation coefficients *r* and associated *p*-values for all raw (mean) data value pairs are given in the figure legend.
(20 KB EPS)

Figure S6 Correlation of the Number of *cis*-Regulatory Elements in the 500-Nucleotide Gene Upstream Promoter Regions with the Breadth of Differential Gene Expression Response for Different Thresholds of Differential Expression Parameters (Fold Up-regulation or Down-regulation and *s*, see Methods) Shown are the mean values and the SEM. Correlation coefficients, *r*, and associated *p*-values are given for all raw data pairs. Note that for more stringent threshold values (e.g., 4-fold versus 2-fold), fewer genes are considered differentially expressed leading to lowered breadth of response values.
(17 KB EPS)

Figure S7 Mean Gene Length, Intron Length (All Intronic Segments per Genes Added Up), and Number of Introns of Genes as a Function of Their Expression Levels Measured by the Median Rank of Gene Expression Levels across All Control Experiments and Binned into Bins of Width 1,000 (31 KB EPS)

Table S1 Set of 43 AtGenExpress Treatment-Control Gene Expression Profiling Experiments Used in the Analysis (25 KB XLS)

## Abbreviations

FDR: false discovery rate
GO: Gene Ontology
nr: nonredundant
RMA: Robust Microchip Analysis
SEM: standard error of the mean
